# Hydrogen Sulfide: Novel Endogenous and Exogenous Modulator of Oxidative Stress in Retinal Degeneration Diseases

**DOI:** 10.3390/molecules26092411

**Published:** 2021-04-21

**Authors:** Panpan Li, Hanhan Liu, Xin Shi, Verena Prokosch

**Affiliations:** Department of Ophthalmology, Faculty of Medicine and University Hospital Cologne, University of Cologne, 50937 Cologne, Germany; panpanlimed@gmail.com (P.L.); hanhan.liu@uk-koeln.de (H.L.); shixinsx123@gmail.com (X.S.)

**Keywords:** oxidative stress, hydrogen sulfide, retinal degeneration diseases, endogenous, exogenous

## Abstract

Oxidative stress (OS) damage can cause significant injury to cells, which is related to the occurrence and development of many diseases. This pathological process is considered to be the first step to trigger the death of outer retinal neurons, which is related to the pathology of retinal degenerative diseases. Hydrogen sulfide (H_2_S) has recently received widespread attention as a physiological signal molecule and gas neuromodulator and plays an important role in regulating OS in eyes. In this article, we reviewed the OS responses and regulatory mechanisms of H_2_S and its donors as endogenous and exogenous regulators in retinal degenerative diseases. Understanding the relevant mechanisms will help to identify the therapeutic potential of H_2_S in retinal degenerative diseases.

## 1. Introduction

### 1.1. Oxidative Stress in Human and Diseases

“They can’t survive without oxygen, but at the same time, oxygen will endanger their health”, which is an interesting paradoxical connection between aerobic organisms and oxygen [1]. Almost all eukaryotic cells produce reactive oxygen species (ROS) and reactive nitrogen species (RNS). Appropriate amounts of ROS and RNS are responsible for regulating several physiological processes, such as proliferation, migration, differentiation, and metabolism [2]. However, excessive ROS and RNS may react with many biologically important molecules, including carbohydrates, lipids, proteins, and nucleic acids, and cause structural damage. This damage is often referred to as oxidative stress (OS) [3]. All animal species have to fight against various stressors that disrupt their homeostasis and respond to OS, which may be exacerbated due to environmental factors (such as pollution, radiation, consumption of certain drugs, cigarette smoke, and consumption of large amounts of alcohol) and physiological factors (such as ischemia, infection, physical or mental stress, and aging). In humans, OS may cause significant damage to cells and is associated with the occurrence and development of a variety of diseases, including cancer [4,5,6], neurodegenerative diseases (such as Alzheimer’s disease and Parkinson’s disease) [7,8,9,10,11,12], cardiovascular diseases [13,14,15], diabetes [16,17,18], etc. OS is also increasingly recognized as a contributing factor in various pathophysiological changes related to aging.

Therefore, it is crucial to identify specific OS-defense molecules. Many molecules can provide chemical protection against OS, commonly known as antioxidants. In the past decades, antioxidants have become the focus of attention.

### 1.2. Background of Endogenous H_2_S

Hydrogen sulfide (H_2_S) is a kind of transparent toxic gas with a strong peculiar odor of rotten eggs [19]. At present, H_2_S has been recognized as a third gaseous signaling molecule, similar to nitric oxide (NO) and carbon monoxide (CO) [20]. H_2_S synthesized from *L*-cysteine in mammalian tissues can pass through the cell membrane directly without specific transporters. A small part of low-level H_2_S can be converted into low-toxicity compounds through the cytosolic detoxification pathway, while most of them are oxidized and metabolized into sulfate and thiosulfate in mitochondria [21,22]. Within 24 h, these metabolites are excreted through the kidneys, intestines, and lungs to maintain the balance of H_2_S levels in the body [23]. A large amount of sulfide is stored in biomolecule-sulfide adducts; this pool could serve as a buffer of free biological sulfide concentrations due to the reversible nature of sulfide binding [24]. In fact, a large number of investigations reveal that, in most cells, tissues, and biological fluids, free sulfide represents less than 1% of the potentially available sulfide, indicating that endogenous sulfide pools should have large buffering capacities [25]. Physiological effects in response to H_2_S typically display a bimodal response that is dependent on the concentration of H_2_S [26]. Under physiological conditions, low concentrations of endogenous H_2_S are not only nontoxic to cells, but have a protective effect. Precise control of endogenous H_2_S production and metabolism is essential to maintain optimal cell function. Excessive production or lack thereof can lead to pathological changes.

#### 1.2.1. H_2_S Synthetizing Enzymes

H_2_S is created enzymatically in humans via multiple conventional and unconventional pathways, of which three pathways are most prominent. These pathways use different enzymes, namely, cystathionine-beta-synthase (CBS), 3-mercaptopyruvate-sulfurtransferase (3-MST), and cystathionine-gamma-lyase (CSE), to create H_2_S gas.

Cysteine serves as a sulfur source for various types of cofactors, such as coenzyme A, biotin, iron-sulfur (Fe-S) clusters, and the molybdenum cofactor; cysteine also plays a pivotal role in protein structure due to its high reactivity and ability to form disulfides in an oxidative environment [27]. CBS is a pyridoxal-5-phosphate-(PLP-)dependent enzyme. Besides producing H_2_S from cysteine, CBS also catalyzes the condensation reaction of homocysteine [28]. In mammals, CBS mRNA and protein are primarily found in the liver, brain, kidney, and pancreas, not in all tissues [29,30], and are the main producer of H_2_S in the central nervous system (CNS) [31]. CSE is expressed in all tissues, with the highest expression level in the liver and kidney [32,33,34]; it produces H_2_S primarily via α, β-elimination of cysteine, forming pyruvate and ammonia in the process [35], and it predominates in the thoracic aorta, ileum, portal vein, and uterus. 3-MST is involved in cysteine catabolism and initially produces an enzyme-bound persulfide via the desulfuration of 3-mercaptopyruvate [36]. However, most of the H_2_S produced by 3-MST is bound in the form of sulfane sulfur, one of the forms in which endogenous H_2_S is stored [37]. The combination of 3-MST and cysteine aminotransferase (CAT) can produce H_2_S [38], and *D*-cysteine can also be used as a substrate to combine with D-amino acid oxidase (DAO) to produce H_2_S [39]. CAT/ 3-MST is expressed in the CNS [40], while DAO/3MST mainly plays a role in the cerebellum and kidney [41]. Although these enzymes also exist in the surrounding tissues, up to now, only the first three endogenous H_2_S-synthesis pathways have been reported to be involved in retinal tissue, and there is no report on the DAO/3MST pathway in retinal tissue.

#### 1.2.2. H_2_S Generation in Ocular Tissues

In recent years, H_2_S has been found in the mammalian retina, and it has been confirmed that the production of H_2_S in eye tissue depends on four main enzymes: CSE, CBS, 3MST, and CAT [38,42]. It is worth noting that these enzymes have also been shown to exist in different parts of eye tissue, especially the retina [43,44,45]. However, due to the different species and methods used in studies, the distribution and regulation of endogenous H_2_S in ocular tissues are controversial.

As early as 2007, Pong et al. [44] discovered the expression and activity of CBS and CSE in the salamander retina, as well as the expression and activity of CSE in the mouse retina. In fact, endogenous H_2_S production has been found in various tissues of bulls’ eyes, including vitreous humor, cornea, aqueous humor, iris, ciliary muscle, lens, choroid, and retina. The highest level of endogenous H_2_S was detected in the cornea and retina, while expression in the retina and optic nerve was slightly lower. The expression in the lens was relatively low, but it was not present in the vitreous humor. In 2011, Kulkarni et al. [46] studied the endogenous H_2_S-synthesis pathway in the bovine retina by using H_2_S-producing enzyme inhibitors. It was found that CBS or CSE inhibitors, or a combination of CBS and CSE inhibitors, could not completely block the production of endogenous H_2_S in the bovine retina. The results suggested that, in addition to CSE and CBE, other enzymes may also participate in the production of endogenous H_2_S in the bovine retina. In the same year, Mikami et al. [45] detected the expression of endogenous H_2_S-producing enzyme in each layer of the mouse retina by immunohistochemistry. The results showed that 3MST and CAT were expressed in the inner reticulum, outer plexiform layer, inner nuclear layer, outer nuclear layer, and the outer segment of photoreceptor, but the expression of CBS and CSE was not detected, which indicated that the generation of H_2_S might be almost catalyzed by the CAT/3MST pathway in the mouse retina [45]. Subsequently, Gersztenkorn et al. in 2016 [47] further confirmed the expression of CBS, CSE, and 3MST proteins in the mouse retina by Western blot and immunohistochemistry. In 2019, Badiei Alireza et al. confirmed the expression of CBS and CSE transcripts in the canine retina by qRT-PCR. Furthermore, they confirmed the expression of CBS in rod cells, amacrine cells, and horizontal cells, and the expression of CSE in RPE, cone cells, and Müller cells by immunohistochemistry and Western blotting [48]. In our previous study, we also showed that endogenous H_2_S synthesis increased through 3-MST in glaucoma animal models after seven weeks of elevated intraocular pressure. CBS is highly expressed in the early part of the entire life cycle and has a tendency towards retinal-dependent age growth. The activity of CSE can be tracked in the retinas of amphibians and mammals [44]. The 3MST/CAT pathway is the main way to produce H_2_S in the mammalian retina, because both 3MST and CAT are located in retinal neurons [45]. The role of endogenous H_2_S in the retina and its physiological role is unclear. However, strong evidence that retinal-derived H_2_S has various effects on ocular tissues under physiological and pathological conditions, especially caused by its concentration. The abnormal expression of endogenous synthetases and the accumulation of substrates and intermediates can change the level of H_2_S by an order of magnitude, resulting in structural or functional abnormalities in the eyes.

#### 1.2.3. Physiological Functions of H_2_S

H_2_S plays a key role in health as a recognized signal molecule and cell protectant. When the concentration of H_2_S in tissues or cells is excessive, H_2_S is considered to be a toxic substance, since it may cause cytotoxicity by inhibiting mitochondrial cytochrome C oxidase and destroying cell energy production, eventually leading to tissue inflammation or DNA damage [49]. Qu et al. [50] suggested that inhibiting the production of endogenous H_2_S may be a potential neuroprotective strategy for strokes. The steady-state free H_2_S concentration in homogenized mouse cells and tissues is estimated to range from 10–30 nM [51,52,53]. In tissues, when H_2_S is produced at a physiological rate or its concentration is kept within a μM range, it can maintain the physiological functions of cells, such as cell division, DNA repair and metabolism, regulation of protein kinase, regulation of the cell cycle, and organization of the cytoskeleton [40]. The abnormal production and metabolism of H_2_S are related to most neurodegenerative diseases [54]. In the CNS, H_2_S can promote long-term potentiation and regulate intracellular calcium concentration and pH in brain cells. The level of endogenous H_2_S in bovine brain tissue and rat brain homogenization has been estimated at 50 to 160 µmol/L [55,56], concentrations high enough to suggest physiological functions. The antioxidant, antiapoptotic, and anti-inflammatory effects of H_2_S have been found in animal or cellular models of Alzheimer’s disease, Parkinson’s disease, and vascular dementia. In addition, H_2_S protects neurons from glutamic acid-mediated OS or OS by maintaining the pleiotropic effects of the cystine/glutamate antiporter, resulting in increased glutathione (GSH) levels in cells [57]. So far, all of it has not been fully elucidated. It is worth noting that almost all physiological aspects are affected by this gas transmitter.

### 1.3. Exogenous Sources of H_2_S

Up to now, various donors have released exogenous H_2_S, and the common ones are inorganic sulfides (such as NaHS and Na_2_S) and morpholin-4-ium-methoxyphenyl-morpholino-phosphinodithioate (GYY4137). Among them, sulfide salts (Na_2_S and NaHS) are the most widely used donors. These inorganic sulfide sources can generate H_2_S instantaneously when they are dissolved, resulting in a rapid rise in the concentration of H_2_S and then a rapid decline. However, GYY4137 slowly releases low but consistent concentrations of H_2_S. Therefore, some H_2_S biological studies use this feature to simulate the time course of the physiological release of H_2_S in vivo. It has been found that exogenous H_2_S can prolong the life of worms, reduce inflammation, and promote the repair of damaged tissues [58]. Studies have found that a lack of H_2_S or its substrate is associated with strabismus, myopia, cataract, optic atrophy, and retinal detachment [59,60,61]. Its exogenous donors have shown the potential to protect retinal ganglion cells (RGC) from diabetic retinopathy (DR), I/R injury, and *N*-methyl-*D*-aspartate (NMDA)-induced excitotoxicity [45,46,47]. In addition, our previous research found that GYY4137, as a sustained-release H_2_S donor, can effectively protect RGC from different glaucoma damage in vitro and in vivo in a dose-dependent manner [62]. H_2_S donors can regulate intraocular pressure, protect retinal cells, inhibit OS, and reduce inflammation by regulating the function of proteins inside and outside the eye tissue. The potential value of H_2_S against OS in human systems and its existence in mammalian eyes should be taken seriously [46]. This review focuses on the anti-OS effect of H_2_S in retinal degenerative diseases and its potential mechanism, which paves the way for understanding the pathogenesis of retinal degenerative diseases and optimizing the application of H_2_S donors in treatment.

## 2. OS in Retinal Degenerative Disease

OS is caused by the imbalance between ROS and the cellular antioxidant defense system in response to endogenous or exogenous stimuli. In fact, in addition to its role in cancer and diabetes, OS is considered to be key to the pathogenesis of many age-related diseases, such as atherosclerosis, cataracts, and Alzheimer’s disease [63,64,65]. The theory of mitochondrial aging suggests that the accumulation of ROS with age will lead to greater cell damage, thereby linking aging, OS, and apoptosis to mediate cell death [66].

**Figure 1:**
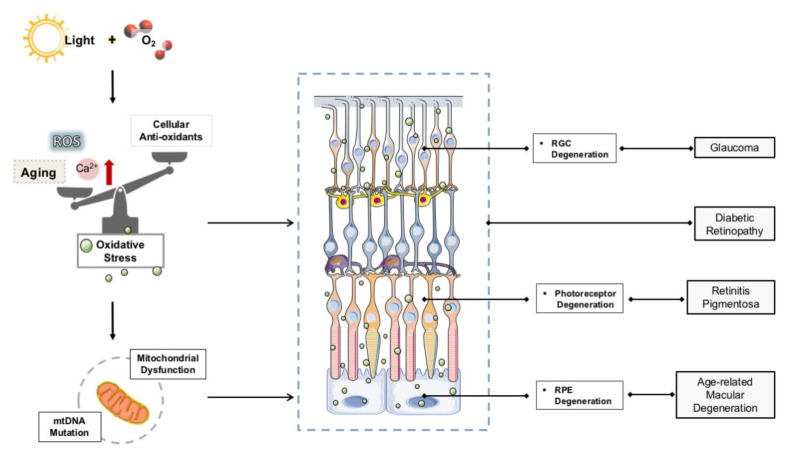
OS in retinal degenerative disease.

The retina is the most metabolically active tissue in the body, and the energy consumption per unit area of tissue is the highest [67]. Photoreceptors and retinal pigment epithelium (RPE) cells are rich in photosensitizers [68,69]. The excessive electromagnetic energy of photons is absorbed by these photosensitizers, and the bonds of other molecules can be destroyed by direct electron exchange or hydrogen exchange, resulting in excessive free radicals [69,70]. In an aerobic environment, oxygen molecules can generate ROS under the excitation of light, such as singlet oxygen, superoxide, and hydrogen peroxide (H_2_O_2_) [71,72]. The increase in the ROS level may lead to OS injuries in photoreceptors and RPE cells [68,73]. In addition, the retina is widely exposed to light and high oxygen pressure, and its photoreceptors are rich in polyunsaturated fatty acids. Both of these substances make the neural retina vulnerable to oxidative damage, leading to mitochondrial dysfunction and mitochondrial DNA (mtDNA) damage. Even in the absence of disease, RPE cells will continue to be exposed to a considerable amount of OS [74]. Therefore, during the aging process, when the antioxidant capacity of RPE cells decreases, the balance between antioxidant and pro-oxidant factors is conducive to an increase in OS [75]. Excessive illumination can lead to photoreceptor degradation, and the cell death of the photoreceptor is irreversible damage caused by an increase in ROS and intracellular Ca^2+^ concentration. These pathological changes can trigger apoptosis [70,76]. These cellular mechanisms are related to the tissue effect of OS. More and more evidence shows that OS injury may be the first step to trigger the death of outer retinal neurons [70,77,78,79,80] and may be related to the pathology of retinal degenerative diseases, as shown in Figure 1. Studies on age-related eye disease (ARED) have shown that vitamin supplementation to control OS can delay disease progression and reduce severity [81].

Age-related macular degeneration (AMD) leads to the loss of central vision, which is the main cause of blindness in the elderly and has an important impact on the quality of life. In AMD, the increases in ROS production, mitochondrial dysfunction, abnormal protein aggregation in the photoreceptor/RPE layer, and increased inflammation lead to OS and damage to RPE cells, which are considered to be key factors in the pathogenesis of AMD [82]. The increase of OS in RPE cells and the retina can lead to inflammation and further enhance the production of ROS in cells and tissues [83,84,85]. There are also studies suggesting that the oxygen-consuming mitochondria in the inner segment play a major role in the OS response of the outer retina [86,87,88].

Glaucoma is an optic neuropathy characterized by loss of RGC. RGC is the key link that transmits signals from the photoreceptor to the optic nerve. The loss of RGC will lead to a gradual loss of vision. In the I/R rat model, we observed that the apoptosis of RGC is related to the OS caused by the accumulation of glutamate [89,90]. Glaucoma also shows a similar pattern of cell damage associated with glutamate accumulation, which can be observed in other neurodegenerative diseases [91,92]. In addition, DNA damage in the trabecular meshwork (TM) by OS can cause anterior chamber aqueous drainage [93], which in turn leads to chronic changes in aqueous and vitreous humors in patients with glaucoma. Therefore, intraocular pressure is the main factor leading to the death of ganglion cells. The direct cause is axonal injury caused by high intraocular pressure, and the indirect cause is a ROS increase caused by RGC death under high intraocular pressure [93]. More interestingly, animal models have shown that age-related mitochondrial defects also play an important role in the pathogenesis of glaucoma [94,95].

DR is one of the most common complications of diabetes. Studies have shown that the activity of antioxidant enzymes in the retinas of diabetic patients is reduced, such as the superoxide dismutase (SOD), GSH reductase, GSH peroxidase, and catalase. In animal models, the administration of antioxidants or overexpression of SOD can protect retinal capillaries from hyperglycemia-induced degeneration. Therefore, ROS is considered to be the main factor involved in the occurrence of DR [94,95,96]. The main site of ROS production in mammalian cells is usually the respiratory transport chain of the mitochondrial inner membrane. Abnormal electron transfer caused by glucose metabolism disorder and excessive ROS triggers mitochondrial dysfunction and membrane potential (ΔΨm) reduction, which together cause mtDNA damage. mtDNA damage induces the synthesis of impaired respiratory chain proteins, thereby increasing the production of ROS, which can activate the nuclear factor-κB (NF-κB) transcription factor, leading to the production of NO and proinflammatory cytokines. Superoxide production has been shown to be increased by uncoupling NO synthase. These superoxides react with NO to form peroxynitrite, which ultimately leads to cell damage and OS [97]. OS directly or indirectly induces inflammatory mediators, leading to retinal cell damage and the subsequent development of the pathogenesis of DR [98].

Retinitis pigmentosa (RP) is the most common degenerative photoreceptor hereditary eye disease characterized by progressive vision loss. Photoreceptors are composed of rod cells (about 95%) and cone cells (only 5%). The main energy comes from the oxidative metabolism of fatty acids. The cytotoxic effect due to the excessive oxygen content caused by a reduction in rod cells may lead to cone cell degeneration in the retina; therefore, OS damage is considered to be the primary cause of cone cell apoptosis and progressive vision loss [99]. OS is one of the most common causes of RP [100] and has also been reported to be associated with other eye diseases, such as AMD [101]. Some studies have confirmed that high levels of ROS in RPE and fatty acids are one of the molecular targets of eye diseases; the occurrence of eye diseases may be due to OS potentially altering transduction pathways and gene expression.

ROS, mainly peroxide and sulfenylated proteins, are inactivated by a variety of antioxidant mechanisms, which include compounds that directly react with ROS (e.g., GSH and ascorbate) and a complement of enzymes that either dismute ROS, e.g., SOD and catalase (Cat), or shuttle electrons from nicotinamide adenine dinucleotide phosphate (NADPH) to the oxidant or oxidized protein [102]. Many molecules can provide chemical protection against OS, commonly known as antioxidants. In the past decades, antioxidants have become a focus of attention. In the CNS, the use of antioxidants as complementary therapy seems promising to reduce nerve injury and improve response. As a kind of physiological signal molecule, H_2_S has been widely studied recently. Some studies have shown that H_2_S can be used as a neurotransmitter to regulate synaptic activity [103]. H_2_S protects neurons from OS damage by increasing the level of GSH, the main antioxidant in cells [104]. Referring to the antioxidant effect of the H_2_S donor on neurons, H_2_S as a gas neuromodulator also plays an important role in regulating OS in the eyes.

## 3. Effects of Endogenous H_2_S on OS in Retinal Neurons

Compared with exogenous antioxidants, endogenous antioxidants like GSH are much more promising, because they are our systematic scavengers with no additional side effects. At present, endogenous information molecules such as CO, NO, and H_2_S are receiving more and more attention. H_2_S itself is not a major cellular antioxidant, but H_2_S can increase GSH by enhancing the activity of γ-glutamylcysteine synthase, thereby protecting cells from OS induced by glutamate and indirectly playing a strong antioxidant role. Earlier studies have shown that the cytoprotective function of H_2_S involves its role in neuronal antioxidant defense.

GSH is one of the main sources of antioxidants in the retina. The Young-Jung Roh [105] et al. studies suggested that GSH depletion may cause unregulated OS to the cells in the retina and indeed increased cell death in the mouse retina. H_2_S production is driven by CSE and CBS, the key enzymes that also drive the transsulfuration pathway (TSP) necessary for GSH production. Badiei et al. [91] revealed that CSE and CBS are expressed in the canine, nonhuman primate (NHP) and human retinas. In vitro silencing of CBS can reduce GSH level [106]. The expression of CBS protein in the synaptic layer of the retina increases with age [43], which is consistent with its role as a key enzyme in homocysteine metabolism because homocysteine levels also tend to increase with age [107], and it may contribute neuronal activity through the production of GSH and H_2_S [48], suggesting that CBS has a protective effect on oxidative damage caused by aging. Compared with CBS, CSE levels did not increase with age. Since CBS can actively produce H_2_S in the retina [108] and brain [109], the antioxidant activity of H_2_S in the retina may be related to the age-dependent increase in CBS [104,110]. Compared with CSE, CBS has played a more active role in the production of H_2_S. RPE and Müller cells produce GSH and H_2_S by CSE to help regulate the immune and inflammatory response of the retina, thereby protecting retinal nerve cells from OS [48]. However, H_2_S enhances the transport of cysteine to increase GSH production to redistribute the localization of GSH to mitochondria, which protects the body against oxidative damage [109]. H_2_S plays roles in antiapoptosis [62,111], anti-inflammatory and OS-suppressing activity [112,113]. These enzymes have potential protective effects on retinal redox [45,114]. Sulfur signaling is not limited to a “one and done” process as the sulfur can be transferred from the protein (which restores its original function) to another thiol such as cysteine or GSH and recycled [115]. H_2_S may play an antioxidant and neuroprotective role in the retina [62,76,104].

H_2_S can protect retinal neurons from light-induced degeneration by regulating Ca^2+^ influx. When retinal photoreceptor cells were exposed to light, intracellular Ca^2+^ concentration is reduced to 10 nm, which activated the 3MST / CAT pathway to produce H_2_S. In the dark, the concentration of Ca^2+^ increased to 600 nm, resulting in the cessation of H_2_S production. H_2_S can protect photoreceptor cells from the effects of apoptosis and OS in retinal cells by activating the vacuolar H+-ATPase (V-ATPase) in horizontal cells, which could activate *L*-type calcium channels (VGCC) to maintain the intracellular calcium balance in photoreceptor cells. Excessive illumination leads to photoreceptor degradation, and its death is irreversible damage caused by ROS and intracellular calcium concentration. Under excessive illumination conditions, endogenous H_2_S regulation of Ca^2+^ may fail and lead to photoreceptor cell degeneration. Even in this case, the degradation of photoreceptors was inhibited by the administration of an H_2_S donor. Under strong light, the increase in the number of TUNEL- and 8-hydroxy-2′-deoxyguanosine-positive cells was suppressed by H_2_S administration [45]. Enhancing the 3MST/CAT pathway or giving H_2_S may have clinical benefits for retinal cell degeneration diseases.

**Figure 2:**
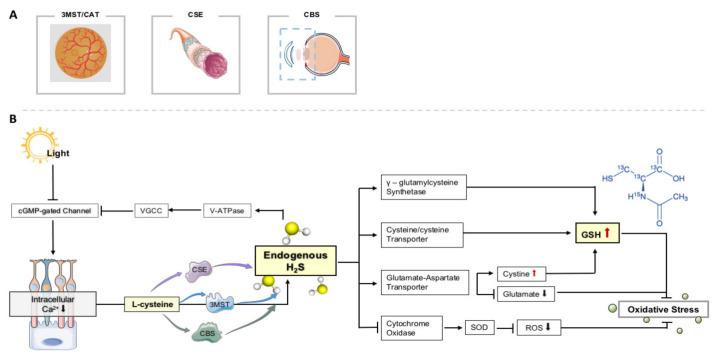
Endogenous H_2_S on OS in retinal neurons. (**A**) The production of H_2_S in eye tissue depends on three main channels. (**B**) Effects of endogenous H_2_S on OS in retinal neurons.

Kimura et al. [104] showed that H_2_S can increase the production of GSH to protect cells from OS by enhancing the cysteine/cysteine transporters and redistributing GSH to mitochondria. In addition, the production of H_2_S in mitochondria may directly inhibit OS. In mitochondria, H_2_S plays a protective role by inhibiting the activity of cytochrome oxidase, upregulating the level of SOD and downregulating the level of ROS after I/R. H_2_S also plays a neuroprotective role by increasing the production of GSH and regulating the transport of CSE to mitochondria and the supply of ATP during hypoxia, as shown in Figure 2.

The glutamate–aspartate transporter in retinoblasts is also involved in maintaining GSH levels [116]. Microglia are the main immune cells in the retina. The aging changes that occur in aging microglia may endow the retina with age-dependent disease vulnerability [117,118]. Glutamate can enhance neurodegeneration induced by cytokines by activating microglia [119]. Glutamate excitotoxicity and associated endoplasmic-reticulum stress conditions are caused by a cascade of events that interfere with the operation of the glutamatergic system, which always lead to microglia activation and inflammation. However, H_2_S therapy may alleviate the above-mentioned stress conditions, and its potential therapeutic target may be the regulation of retinal neurotransmission transporters related to glutamate excitotoxicity. Glutamate shares the same amino acid transporter with cystine, and it competes with cystine for transport into cells [120]. Therefore, in the process of glutamate excitotoxicity mediated by OS, elevated extracellular glutamate inhibits the transport of cystine [57]. Cystine is the primary source of intracellular cysteine necessary for GSH synthesis. The decrease in cysteine input leads to the decrease in GSH synthesis. It seems that H_2_S can restore glutamic acid-inhibited cystine input. In short, H_2_S treatment may reduce glutamate excitotoxicity, endoplasmic-reticulum stress and microglia activation, which are related to OS.

## 4. Effects of Exogenous H_2_S on OS in Retinal Neurons

In previous studies by different research groups, including us, exogenous donors have shown therapeutic potential in a variety of retinal diseases. The neuroprotective effect of H_2_S may be related to the inhibition of glial cell activation, inhibition of pro-inflammatory pathway, inhibition of inflammatory response, reduction of OS, and downregulation of retinal autophagy [62,111,121].

**Figure 3:**
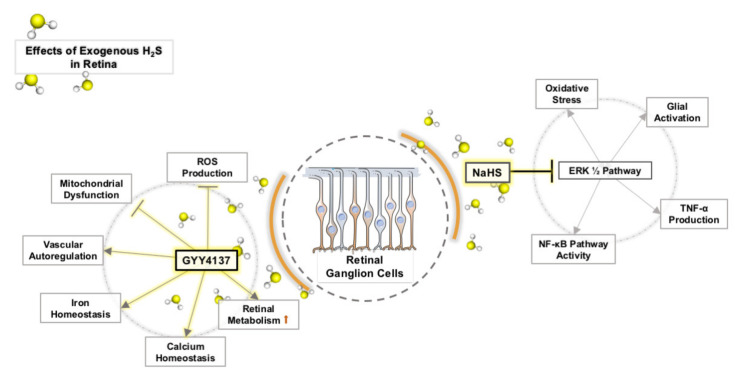
Effects of exogenous H_2_S on OS in retinal neurons.

NADPH oxidase is the main source of ROS and plays a key role in the aggravation of retinal OS in glaucoma models [122]. Results showed that the activity of NADPH oxidase in the retinas of experimental glaucoma rats was inhibited by exogenous donor NaHS [111]. Mitochondria maintain basic energy metabolism mainly through oxidative phosphorylation. The cellular ATP content and oxygen-consumption capacity produced by the mitochondrial respiratory chain can reflect the integrity of mitochondrial function. Mitochondrial dysfunction even contributes to the accumulation of ROS, which aggravates OS. NaHS treatment can effectively restore mitochondrial function in RGC in experimental glaucoma [111]. NaHS treatment in the retina of streptozotocin-induced diabetic rats also attenuated mitochondrial dysfunction [123]. Huang et al. [121] have shown that NaHS can inhibit the activity of the erk1/2 pathway, inhibit the activity of NADPH oxidase, and inhibit the activation of glial cells, as shown in Figure 3.

GYY4137 is a sustained-release H_2_S donor. We observed that 100 mM H_2_O_2_ induced significant OS in retinal explants of the experimental glaucoma model. However, the addition of 100 nM and 10 µM GYY4137 can significantly increase the survival rate of RGC, although 1 nM GYY4137 has no protective effect on 100 mM H_2_O_2_. At a certain concentration, GYY4137 can reduce the damage of OS to RGCs (Figure 3). Our study found that GYY4137 has a concentration-dependent neuroprotective effect on glaucomatous retinal damage caused by OS in vitro. Generally, a higher concentration is required to preserve RGCs, and the optimal concentration is 100 nM [62].

Synuclein is a small protein family, including α (SNCA), β (SNCB), and c (SNCG) synuclein [124,125], which are involved in various neurodegeneration effects of the CNS. The misfolding and aggregation of SNCA is directly related to the activation of microglia, and the subsequent inflammation and OS lead to neurodegeneration [126]. Synuclein is present in the retina and optic nerve [127]. Under physiological conditions, SNCB is considered to be a physiological inhibitor of SNCA and an antiaggregation agent with neuroprotective effects. However, the protective effect of SNCB is exerted in a dose-dependent manner [124], which means that the overexpression and accumulation of SNCB will increase OS and inflammation and promote cell apoptosis [128,129]. SNCB was found to form toxic cytoplasmic inclusion bodies in a manner similar to SNCA and has similar toxic mechanisms, including vesicle transport damage and OS induction [130]. H_2_S is involved in some pathophysiological processes that interact with SNCB, such as microglia activation, p53-mediated apoptosis, inflammatory response, and free radical reaction [121,131,132,133].

Our research shows that increasing exogenous H_2_S can effectively downregulate rat retinal SNCB and has a protective effect on retinal neurons [134]. Therefore, removing the pathogenic SNCB or reducing its abundance may be an effective way to rescue neurons and prevent the progression of glaucoma. H_2_S protects retinal neurons from excessive OS [45,63,123], which further confirms that the reduction of OS may be involved in the neuroprotective effect of H_2_S supplementation on RGCs in glaucoma.

In our previous studies, we have also revealed that exogenous H_2_S activates the pathways related to OS, such as iron regulation, ROS clearance, and mitochondrial homeostasis and function [135]. Vitamin C also plays an important role as an antioxidant in the retina and brain [136]. Vitamin C is mainly transported across the blood–retinal barrier (BRB) as dehydroascorbic acid (DHA), mediated through facilitative glucose transporters (GLUT)1, and accumulates as ascorbic acid (AA) in the rat retina [137,138]. The transporters in the BRB play a role in exogenous H_2_S donor delivery. Because the BRB regulates the transfer of small molecules from the eye outwards (intravitreal injection) and inwards into the eye (systemic, topical (trans-scleral route), subconjunctival, and suprachoroidal administration) [139]. Although understanding of the active drug transport in the BRB is still far from complete, there is evidence for the role of some transporters in drug delivery, these transporters include MRP1 [140,141,142,143], MRP2 [144,145], MRP5 [141], P-gp [142,146,147], and BCRP [145]. DHA is a transportable form across the inner BRB; the transport and reduction of DHA in Müller cells plays an important role in the maintenance of a healthy retina [148]. Slc2a1 promotes DHA to be transported through the inner BRB [149]. The downregulation of Slc2a1 levels leads to a corresponding downregulation of vitamin C transport. Downregulation of vitamin C transport will affect the antioxidant properties of the retina, which will contribute to neuronal death under OS [150]. We have observed that exogenous H_2_S can increase the transport of vitamin C by restoring the level of Slc2a1, thereby enhancing the antioxidant properties of the retina [130]. The metabolism and regulation of iron are crucial in mammals. Excessive iron may catalyze the formation of highly active hydroxyl radicals and ultimately induce the accumulation of ROS [151,152]. Glrx3 is a single-mercapto GSH that uses the reducing power of GSH to maintain and regulate the redox state of cells, which is necessary for the assembly of Fe-S clusters [153,154]. Meanwhile Glrx3 can form a (2Fe-2S) cluster bridged dimer, transfer Fe-S clusters to receptor proteins [155,156], and regulate cellular iron homeostasis [157,158]. In our experiment, we found that H_2_S can significantly upregulate Glrx3 [135]. Overexpression of Glrx3 can make up for the lack of other reduction equivalents [158]. Most of the iron in the human body is contained in the protoporphyrin ring of heme [152]. Heme degradation is very important in maintaining iron homeostasis and preventing its cytotoxicity and OS [159]. Cyb5r3 is the main reductase in mitochondria and heme reductase, which can recover oxidized heme (Fe^3 +^) to reduced form (Fe^2 +^) [160]. H_2_S plays a reducing role similar to that of Cyb5r3 in cycling oxidized heme to the reduced state [135]. Under the conditions of I/R, H_2_S can effectively inhibit oxidative phosphorylation and limit the utilization of glucose as an energy source, thereby increasing intracellular oxygen tension during ischemia and limiting the production of ROS during reperfusion. H_2_S promotes the use of ketone bodies as an alternative energy source and maintains the production of ATP. Metabolic stress usually exhausts the elasticity of neurons and leads to the loss of neuronal cells. Obviously, H_2_S enhances the ability of retinal neurons to resist I/R-induced metabolic stress [135].

## 5. Conclusions

It is well known that OS-mediated inflammatory processes in the eyes of susceptible hosts can lead to the initiation of subtle pathological changes, which trigger a degenerative and inflammatory cascade in the retina. H_2_S and its donors, as a regulator of OS in retinal degenerative diseases, can alleviate retinopathy by inhibiting the harmful effects of OS. It seems that further research will greatly improve our understanding of the pathophysiological mechanisms of retinal degenerative diseases and the potential for the treatment of retinal diseases associated with H_2_S.

## Data Availability

Not applicable.
